# Pulmonary Cryptococcosis With Suspected Central Nervous System Involvement in a Patient With Sarcoidosis and CD4 Lymphopenia: A Case Report

**DOI:** 10.7759/cureus.111543

**Published:** 2026-06-26

**Authors:** Sara Almaradweh, Zonaira Gul

**Affiliations:** 1 Medicine, Avalon University School of Medicine, Willemstad, CUW; 2 Infectious Diseases, Beckley Appalachian Regional Healthcare, Beckley, USA

**Keywords:** amphotericin b nephrotoxicity, cd4 lymphopenia, cell-mediated immunity, cryptococcal antigen, immunocompromised host, opportunistic infection, pulmonary cryptococcosis, recurrent herpes zoster, sarcoidosis, suspected central nervous system involvement

## Abstract

Sarcoidosis is a multisystem granulomatous disorder associated with immune dysregulation and impaired cell-mediated immunity, which may increase susceptibility to opportunistic infections, including cryptococcosis. The clinical and radiological overlap between sarcoidosis progression, neurosarcoidosis, and cryptococcal infection can create a significant diagnostic challenge, particularly when neurological symptoms are present. We present a 50-year-old male with a 15-year history of pulmonary sarcoidosis and CD4 lymphopenia who developed pulmonary cryptococcosis with subsequent symptoms concerning for central nervous system (CNS) involvement after self-discontinuation of antifungal therapy. His clinical course was notable for neck stiffness, photophobia, headaches, cognitive slowing, positive serum cryptococcal antigen testing, bronchoscopy confirming *Cryptococcus* species, and recurrent herpes zoster, suggesting impaired cell-mediated immunity. Cerebrospinal fluid culture showed no growth after antifungal therapy had already been initiated, and CNS involvement was considered clinically suspected rather than microbiologically confirmed. Treatment was complicated by acute kidney injury attributed in part to amphotericin B-associated nephrotoxicity, requiring transition to fluconazole consolidation therapy. This case underscores the importance of maintaining a high index of suspicion for opportunistic infections in patients with sarcoidosis who develop atypical, progressive, or neurological manifestations. Early cerebrospinal fluid evaluation, including cryptococcal antigen testing when clinically indicated, and careful infectious disease assessment before escalation of immunosuppressive therapy are essential to avoid diagnostic delay and guide appropriate treatment.

## Introduction

Sarcoidosis is a multisystem inflammatory disorder characterized by non-caseating granulomas in affected organs, most commonly the lungs and intrathoracic lymph nodes. The disease presents a striking immunological paradox: robust, localized type 1 helper T-cell responses at sites of granulomatous inflammation coexist with peripheral anergy and impaired cell-mediated immunity [1,2]. This systemic immune dysfunction renders patients susceptible to opportunistic infections even without traditional risk factors such as HIV infection or intensive immunosuppressive therapy. Notably, cryptococcosis occurs in one-third of sarcoidosis patients without any immunosuppressive treatment, emphasizing the intrinsic immune defects underlying this association [3].

*Cryptococcus neoformans*, an encapsulated yeast ubiquitous in soil and avian droppings, poses a significant threat to this vulnerable population. While immunocompetent hosts typically clear or contain the organism, patients with compromised T-cell function are at risk for severe pulmonary disease or disseminated infection, particularly life-threatening meningoencephalitis. Among HIV-seronegative patients with cryptococcosis, sarcoidosis accounts for 2.9% of cases, with extra-thoracic sarcoidosis and corticosteroid therapy representing independent risk factors [3]. Sarcoidosis was the most frequently associated condition with central nervous system (CNS) cryptococcosis in one recent series, accounting for 31% of cases [2].

The co-occurrence of sarcoidosis and cryptococcosis presents a profound diagnostic and therapeutic challenge. Corticosteroids remain first-line therapy for symptomatic sarcoidosis to suppress granulomatous inflammation; however, this treatment further impairs host immune defenses, potentially unmasking or exacerbating latent fungal infection. The clinical and radiological features of cryptococcal infection frequently mimic sarcoidosis progression, presenting with worsening pulmonary nodules, lymphadenopathy, and constitutional symptoms [4,5].

This diagnostic mimicry carries serious consequences. In 43% of reported cases, cryptococcal meningitis was initially misdiagnosed as neurosarcoidosis, resulting in treatment delays that doubled the rate of unfavorable outcomes (41% versus 21%) [1]. Overall mortality from cryptococcal meningitis in sarcoidosis patients reaches 19-32%, predominantly attributable to delayed diagnosis [1,3].

This case report illustrates the intricate relationship between these two conditions and underscores the critical importance of rigorous infectious disease evaluation before initiating or escalating immunosuppressive therapy in patients with sarcoidosis presenting with atypical or progressive manifestations.

## Case presentation

A 50-year-old male with a 15-year history of multisystem sarcoidosis presented with progressive respiratory decline, constitutional symptoms, and subsequent symptoms concerning for CNS involvement from cryptococcosis. His sarcoidosis was initially diagnosed in 2010 after he developed a persistent dry cough that progressively worsened. After tuberculosis and malignancy were excluded, bronchoscopy with transbronchial biopsy demonstrated non-caseating granulomas consistent with pulmonary sarcoidosis. His disease primarily involved the lungs and intrathoracic lymph nodes. He also had chronic tachycardia, with a baseline heart rate of approximately 105-110 beats per minute, which was attributed clinically to possible cardiac sarcoidosis involvement.

His past medical history was also significant for type 2 diabetes mellitus, hypertension, chronic obstructive pulmonary disease, gastroesophageal reflux disease, and remote meningitis at age 10. He was a lifelong non-smoker. His immune history was notable for recurrent herpes zoster infections, occurring up to four times. His sarcoidosis treatment had included prednisone 5 mg four times daily, which was later tapered because of type 2 diabetes mellitus with a hemoglobin A1c of 7.0%. He was subsequently transitioned to dupilumab as steroid-sparing therapy; no other indication was documented in the available records. Dupilumab was discontinued in December 2025 after cryptococcal infection was identified.

The patient reported that sarcoidosis had significantly affected his daily function and work capacity. He previously worked in a field-based regional adoption role that required home visits, but progressive dyspnea forced him to transition to a sedentary desk-based position. He described becoming short of breath more easily and having difficulty maintaining his prior level of activity. He also reported concentration difficulties that he attributed to his sarcoidosis.

**Figure 1:**
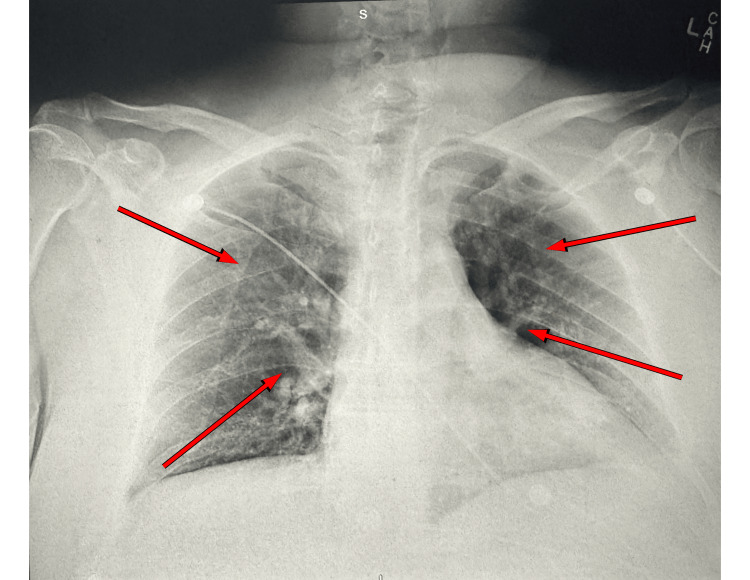
Chest radiograph obtained during follow-up in January 2026 demonstrating mild diffuse bilateral increased interstitial markings, highlighted by arrows. No focal consolidation or pleural effusion was identified.

In late 2025, he developed progressive dyspnea, severe fatigue, low-grade fever, night sweats, chest pain, and unintentional weight loss of approximately 25 pounds over six weeks. In November 2025, bronchoscopy was performed because of worsening respiratory symptoms. Bronchoalveolar lavage fluid culture and/or tissue biopsy grew *Cryptococcus* species, establishing the diagnosis of pulmonary cryptococcosis. Earlier chest computed tomography had demonstrated pulmonary nodules and intrathoracic lymphadenopathy consistent with his known sarcoidosis.** **Chest radiography obtained during follow-up in January 2026 demonstrated nonspecific, mild, diffuse increased interstitial markings without focal consolidation, pleural effusion, or radiographic evidence of active tuberculosis (Figure 1).

Serum cryptococcal antigen testing was positive in late December 2025. Initial laboratory evaluation demonstrated leukopenia, with a white blood cell count of 3.71 × 10³/µL, below the reference range of 4.0-11.0 × 10³/µL. Hemoglobin was 11.1 g/dL, below the adult male reference range of 13.5-17.5 g/dL. His CD4 count was decreased to 284 cells/µL, compared with a reference range of 500-1,500 cells/µL. Immunoglobulin testing showed preserved humoral immunity, with IgG at 808 mg/dL, within the reference range of 700-1,600 mg/dL; IgM at 72 mg/dL, within the reference range of 40-230 mg/dL; and IgA at 241 mg/dL, within the reference range of 70-400 mg/dL. Baseline renal function was normal, with an estimated glomerular filtration rate of 102 mL/min/1.73 m². HIV serology was negative (Table 1).

**Table 1:** Pertinent laboratory, microbiological, CSF, and renal function findings. CSF: cerebrospinal fluid; VDRL: Venereal Disease Research Laboratory test.

Test	Patient result	Normal reference range	Interpretation
White blood cell count	3.71 × 10³/µL	4.0-11.0 × 10³/µL	Low
Hemoglobin	11.1 g/dL	13.5-17.5 g/dL	Low
CD4 count	284 cells/µL	500-1,500 cells/µL	Low
IgG	808 mg/dL	700-1,600 mg/dL	Normal
IgM	72 mg/dL	40-230 mg/dL	Normal
IgA	241 mg/dL	70-400 mg/dL	Normal
Estimated glomerular filtration rate	102 mL/min/1.73 m²	>90 mL/min/1.73 m²	Normal baseline renal function
Baseline creatinine	~0.7 mg/dL	0.7-1.3 mg/dL	Normal
Peak creatinine after amphotericin B exposure	3.6 mg/dL	0.7-1.3 mg/dL	Acute kidney injury
Creatinine at the later January 2026 presentation	2.6 mg/dL	0.7-1.3 mg/dL	Improving but elevated
Creatinine after IV fluids	1.26 mg/dL	0.7-1.3 mg/dL	Improved
HIV serology	Negative	Negative	No evidence of HIV infection
Serum cryptococcal antigen	Positive	Negative	Abnormal
Serial cryptococcal antigen titers	Not available	—	Not available for treatment-response monitoring
CSF opening pressure	Not documented	—	Not available
CSF white blood cell count	4 cells/µL	0-5 cells/µL	Within normal range
CSF glucose	112 mg/dL	40-75 mg/dL	High
CSF protein	62 mg/dL	15-45 mg/dL	High
CSF VDRL	Nonreactive	Nonreactive	Negative
CSF Gram stain	No organisms seen	No organisms seen	Negative
CSF culture	No growth	No growth	No growth after antifungal initiation

The patient was initially treated as an outpatient with fluconazole 400 mg daily; however, he self-discontinued the medication because of severe insomnia. He subsequently developed symptoms concerning for CNS involvement, including neck pain and stiffness, photophobia, headaches, cognitive slowing, and visual disturbances described as a halo or blurred effect. He reported that neck extension was particularly painful and that he had difficulty bringing his chin fully to his chest. During hospitalization, his photophobia required the room lights to be turned off. He also described slowed responses and difficulty remembering information, which improved after initiation of antifungal therapy.

The patient was hospitalized from late December 2025 to early January 2026 for 11 days for suspected CNS involvement of cryptococcosis and induction of antifungal therapy. He received intravenous amphotericin B during the admission. His headaches improved after approximately two doses of amphotericin B. Lumbar puncture was performed during hospitalization. Cerebrospinal fluid (CSF) opening pressure was not documented in the available records. The CSF sample was described as less than optimal and predominantly bloody, with many red blood cells present. CSF analysis demonstrated a white blood cell count of 4 cells/µL, glucose of 112 mg/dL, and protein of 62 mg/dL. A cell count differential was not performed because the CSF white blood cell count was less than 6 cells/µL. The CSF Venereal Disease Research Laboratory (VDRL) test was nonreactive. Gram stain showed no organisms, and CSF culture showed no growth. These CSF findings were interpreted cautiously because antifungal therapy had already been initiated, and the specimen was predominantly bloody and suboptimal for complete analysis. The negative CSF culture was therefore not considered sufficient to confirm or exclude CNS involvement. Formal neuroimaging findings were not available for detailed reporting. Serial cryptococcal antigen titers were not available for treatment-response monitoring.

Treatment was complicated by acute kidney injury attributed in part to amphotericin B-associated acute tubular injury, with hypokalemia requiring potassium supplementation. His baseline creatinine was approximately 0.7 mg/dL, with an estimated glomerular filtration rate of 102 mL/min/1.73 m². After approximately 10 days of amphotericin B exposure from late December 2025 to early January 2026, his creatinine was reported to have peaked at 3.6 mg/dL during a subsequent readmission in mid-January 2026. He later presented in late January 2026 with a creatinine of 2.6 mg/dL, which improved to 2.21 mg/dL and then 1.26 mg/dL after intravenous fluid resuscitation. Nephrology assessment favored multifactorial acute kidney injury due to amphotericin B-associated acute tubular injury with superimposed prerenal azotemia. Because of renal toxicity, amphotericin B was discontinued after approximately 10-11 days of therapy, and he was transitioned to oral fluconazole 400 mg daily for consolidation and maintenance therapy. Flucytosine was not administered during the induction phase. There was no documentation of serial lumbar punctures for intracranial pressure management or ventriculoperitoneal shunt placement.

At outpatient follow-up in late January 2026, the patient reported clinical improvement and was stable on fluconazole 400 mg daily. His renal function was improving after discontinuation of amphotericin B. He continued close follow-up with infectious disease specialists and remained on long-term antifungal therapy. His persistent exertional dyspnea continued to limit his ability to return to field-based work and was attributed primarily to his underlying sarcoidosis.

## Discussion

Diagnostic challenges: The mimicry of neurosarcoidosis

This case powerfully illustrates the diagnostic pitfalls inherent in the co-occurrence of sarcoidosis and cryptococcosis, two conditions that share overlapping clinical and radiological features yet require diametrically opposed therapeutic approaches. Both diseases present with intrathoracic lymphadenopathy, nodular interstitial markings, constitutional symptoms, including weight loss and fatigue, and neurological manifestations. The patient's worsening respiratory symptoms, weight loss, night sweats, and constitutional decline could easily have been attributed to sarcoidosis progression rather than opportunistic infection. The diagnostic trap is well-documented in the literature: cryptococcal meningitis was initially misdiagnosed as neurosarcoidosis in 43% of reported cases, resulting in treatment delays that doubled the rate of unfavorable outcomes (41% versus 21% in patients without delayed diagnosis) [1]. Overall mortality from cryptococcal meningitis in sarcoidosis patients reaches 19-32%, predominantly attributable to delayed diagnosis [1]. In this patient, the November 2025 bronchoscopy provided the critical diagnostic breakthrough, identifying *Cryptococcus* species in lung tissue when respiratory symptoms worsened despite sarcoidosis-directed therapy. This finding underscores the importance of maintaining a high index of suspicion for opportunistic infections in sarcoidosis patients with atypical or progressive manifestations [6,7].

Comparison with published case series

When compared with published case series, this patient's presentation demonstrates both typical and distinctive features. In the CryptOsarc study of 18 sarcoidosis patients with cryptococcosis, the median age was similar to that of our patient, and the site distribution included cryptococcal meningitis in 72%, osteoarticular involvement in 17%, and disseminated infection in 22% [3]. The patient’s progression from pulmonary cryptococcosis to symptoms concerning for CNS involvement resembles the reported pattern of meningeal involvement, although CNS disease was clinically suspected rather than microbiologically confirmed in this case.

Regarding CD4 counts, published series report median values of 145 cells/mm³ (range = 55-1300) in the CryptOsarc cohort and 84-228 cells/mm³ in a systematic review of cryptococcal meningitis complicating sarcoidosis [1,3]. Paradoxically, this patient's CD4 count of 284 cells/µL is higher than the typical range reported in sarcoidosis-cryptococcosis, yet he still developed pulmonary cryptococcosis with symptoms concerning for CNS involvement. This observation aligns with recent findings that sarcoidosis patients with CNS cryptococcosis demonstrate higher CD4 percentages and CD4/CD8 ratios compared to non-sarcoidosis patients (47 ± 12% vs. 22 ± 18%, p = 0.02), suggesting that CD4 lymphopenia alone does not fully explain the sarcoidosis-cryptococcosis association [2]. Qualitative T-cell dysfunction or other immune defects, rather than absolute CD4 depletion, appear to play critical roles in cryptococcal susceptibility [2,8].

Regarding treatment response, the CryptOsarc study reported that 89% of patients (16/18) presented a complete response to antifungal therapy, and after a mean follow-up of six years, no death was attributable to cryptococcosis [3]. This favorable prognosis contrasts with the higher mortality rates seen in other immunocompromised populations and likely reflects the qualitative rather than quantitative nature of immune dysfunction in sarcoidosis.

Clinically relevant features of this case

Several features are clinically notable in this case. First, the patient’s history of recurrent herpes zoster may reflect impaired cell-mediated immunity and could represent a clinical clue to increased susceptibility to opportunistic infection. This pattern of repeated viral reactivation may suggest an underlying immunological vulnerability in patients with sarcoidosis.

Second, the patient was receiving dupilumab, a monoclonal antibody targeting IL-4 receptor alpha, at the time of cryptococcal diagnosis. While dupilumab is generally not associated with increased opportunistic infections, any modulation of the cytokine environment in a patient with existing sarcoid-related lymphopenia may have contributed to immune dysregulation. This case adds to the limited literature on cryptococcosis occurring during biologic therapy for sarcoidosis.

Third, the positive serum cryptococcal antigen with bronchoscopy confirming *Cryptococcus* species and negative CSF cultures raised consideration of either initially localized pulmonary disease or possible *Cryptococcus gattii* involvement rather than* C. neoformans*. However, the possibility of *Cryptococcus gattii* involvement remains speculative because species-level identification was not available. Unlike *C. neoformans*, which primarily targets severely immunocompromised hosts, 50-70% of *C. gattii *infections occur in putatively immunocompetent hosts with subtle T-cell defects.

Fourth, the patient’s possible cardiac sarcoidosis involvement, reflected by chronic tachycardia attributed clinically to sarcoidosis, may be clinically relevant because neurological or cardiac sarcoidosis involvement has been associated with increased risk for severe infections, with odds ratios of 3.36 and 2.65, respectively [9].

Fifth, the patient's classic meningeal symptoms, including neck stiffness, photophobia requiring environmental modification, headaches, and cognitive slowing, are notable because such symptoms occur in only one-quarter to one-third of patients with cryptococcal meningitis. Their presence in this patient raised clinical concern for CNS involvement. However, because CSF culture showed no growth after antifungal therapy had already been initiated and formal neuroimaging findings were unavailable, CNS involvement should be interpreted as clinically suspected rather than confirmed.

Pathophysiology: Potential immune mechanisms

This patient’s susceptibility to cryptococcosis with suspected CNS involvement may reflect a convergence of multiple immunological vulnerabilities, including sarcoidosis-associated immune dysregulation, prior corticosteroid exposure, recurrent herpes zoster, suggesting impaired cell-mediated immunity, and possible unmeasured immune defects. Sarcoidosis embodies an immunological paradox: although it is characterized by intense local granulomatous inflammation driven by robust Th1 responses, patients simultaneously manifest peripheral anergy and impaired systemic cell-mediated immunity [10,11].

Critically, cryptococcosis occurs in one-third of sarcoidosis patients without any immunosuppressive treatment, emphasizing that intrinsic immune defects, rather than iatrogenic immunosuppression alone, predispose to opportunistic infections [3]. The mechanisms underlying this vulnerability remain incompletely understood, but several pathophysiological explanations have been proposed.

First, sequestration of activated T-lymphocytes at sites of granulomatous inflammation may deplete the peripheral T-cell pool, resulting in systemic anergy despite local immune hyperactivity. Second, B-1 cell deficiency or lack of IgM may impair initial antibody responses against cryptococcal polysaccharide capsule [2]. Third, anti-GM-CSF autoantibodies, classically associated with pulmonary alveolar proteinosis, have been identified in 76% of apparently immunocompetent patients with *C. gattii* infection and in 5.4% of sarcoidosis patients [2]. These autoantibodies impair macrophage phagocytic function by preventing GM-CSF-induced STAT5 phosphorylation, representing a plausible mechanism for cryptococcal susceptibility. Because anti-GM-CSF antibody testing was not performed, its role in this patient remains hypothetical and should be interpreted as a possible mechanism rather than a confirmed contributor. The patient’s sarcoidosis-associated T-cell dysfunction and recurrent herpes zoster suggest impaired cell-mediated immunity, which may have contributed to susceptibility to cryptococcal infection and possible progression.

Additionally, cryptococcal infection may preferentially engage immune-privileged body compartments, including the CNS, tumor microenvironments, and certain granuloma types [8]. This tropism suggests that opportunistic pathogens exploit immunodeficient anatomical locations even when systemic immunity appears relatively preserved.

The steroid legacy also warrants consideration. Although prednisone was discontinued due to diabetes, historical corticosteroid use in a patient with pre-existing lymphopenia may have contributed to long-term immune remodeling. Extra-thoracic sarcoidosis and corticosteroids are independent risk factors for cryptococcosis in multivariate analysis [3]. The patient’s history of recurrent shingles may support underlying impairment in cell-mediated immunity.

Treatment considerations

Management of this patient required careful navigation between treating a life-threatening fungal infection and managing a chronic inflammatory disease; a therapeutic "tightrope walk" with significant risks on both sides.

Recommended treatment approaches for severe pulmonary or disseminated cryptococcosis include treating severe pulmonary or disseminated cryptococcosis as CNS disease, with preferred induction therapy of liposomal amphotericin B 3-4 mg/kg daily plus flucytosine 25 mg/kg four times daily for two weeks, followed by consolidation with fluconazole 400-800 mg daily for eight weeks, then maintenance with fluconazole 200 mg daily for six to 12 months. For mild isolated pulmonary disease without CNS involvement, fluconazole 400 mg daily for six to 12 months is appropriate [12].

This patient’s treatment course differed from guideline-preferred recommendations in several respects. First, flucytosine was not administered as part of the induction regimen. The combination of amphotericin B plus flucytosine produces more rapid fungicidal activity and enables abbreviation of the amphotericin B course, thereby minimizing toxicity. The absence of flucytosine may warrant close monitoring for relapse.

Second, amphotericin B was discontinued after approximately 11 days due to treatment-limiting nephrotoxicity and severe hypokalemia [12]. The specific amphotericin B formulation was not documented in the available records. The patient was transitioned to oral fluconazole 400 mg daily for consolidation and maintenance therapy.

Third, the patient's self-discontinuation of initial outpatient fluconazole due to severe insomnia may have contributed to ongoing disease activity and subsequent concern for CNS involvement. This underscores the importance of patient education regarding antifungal adherence and proactive management of medication side effects.

To treat the infection, dupilumab was discontinued immediately upon diagnosis. This created a secondary risk, i.e., the potential for immune reconstitution inflammatory syndrome (IRIS), where a recovering immune system mounts an exaggerated inflammatory response against fungal antigens. The patient's clinical improvement without apparent IRIS was reassuring, although ongoing follow-up remained important.

Prognosis and long-term outcomes

Despite pulmonary cryptococcosis with clinical concern for CNS involvement, the prognosis for sarcoidosis patients with cryptococcosis is generally favorable with appropriate antifungal treatment. In the CryptOsarc study, 89% of patients (16/18) presented a complete response to antifungal therapy, and after a mean follow-up of six years, no death was attributable to cryptococcosis [3]. This favorable outcome contrasts with the higher mortality rates seen in other immunocompromised populations.

However, outcomes are significantly worse when the diagnosis is delayed. The rate of unfavorable outcome in patients with delayed diagnosis was 41% compared to 21% in patients diagnosed promptly [1]. This patient's relatively early diagnosis, prompted by bronchoscopy when respiratory symptoms worsened, likely contributed to his favorable clinical trajectory.

Clinical implications

This case reinforces several critical clinical implications. First, a high index of suspicion for opportunistic infections must be maintained in sarcoidosis patients presenting with atypical or progressive manifestations, even in the absence of immunosuppressive therapy [3,10,13].

Second, early cryptococcal antigen testing should be performed in sarcoidosis patients with meningitis or neurological symptoms. CSF cryptococcal antigen testing has 93% sensitivity and should not be omitted based on negative serum cryptococcal antigen results [1,2].

Third, recurrent herpes zoster may serve as a clinical marker of T-cell dysfunction and heightened susceptibility to opportunistic pathogens, warranting increased vigilance in affected patients.

Fourth, rigorous infectious disease evaluation should precede initiation or escalation of immunosuppressive therapy in sarcoidosis [9,12].

Fifth, patient education regarding antifungal adherence is critical to prevent treatment interruption and disease progression.

Limitations

This case is limited by the absence of documented CSF opening pressure, lack of documented CSF cryptococcal antigen testing, lack of serial cryptococcal antigen titers, negative CSF culture after antifungal therapy had already been initiated, unavailable formal neuroimaging findings, and lack of species-level identification. Therefore, CNS involvement was considered clinically suspected rather than microbiologically confirmed, and proposed mechanisms such as anti-GM-CSF antibody involvement or possible *Cryptococcus gattii *infection should be interpreted as hypotheses rather than confirmed findings.

## Conclusions

This case illustrates the complex diagnostic overlap between sarcoidosis and cryptococcal infection, two conditions that can share pulmonary, constitutional, and neurological manifestations but require different therapeutic approaches. In this patient, pulmonary cryptococcosis was confirmed by bronchoscopy and positive serum cryptococcal antigen testing, while CNS involvement was clinically suspected rather than microbiologically confirmed. The case highlights the importance of maintaining a high index of suspicion for opportunistic infection in patients with sarcoidosis, particularly those with CD4 lymphopenia, recurrent herpes zoster, corticosteroid exposure, or atypical progressive symptoms. Early infectious disease evaluation, CSF testing, including cryptococcal antigen when clinically indicated, and cautious interpretation of incomplete diagnostic data are essential to avoid diagnostic delay and guide appropriate therapy.
